# Evaluating the Potential Use of Serotonergic Psychedelics in Autism Spectrum Disorder

**DOI:** 10.3389/fphar.2021.749068

**Published:** 2022-01-27

**Authors:** Athanasios Markopoulos, Antonio Inserra, Danilo De Gregorio, Gabriella Gobbi

**Affiliations:** ^1^ Neurobiological Psychiatry Unit, Department of Psychiatry, McGill University, Montreal, QC, Canada; ^2^ McGill University Health Centre, McGill University, Montreal, QC, Canada

**Keywords:** autism, psychedelics, behaviour, neurobiology, LSD

## Abstract

Recent clinical and preclinical evidence points towards empathogenic and prosocial effects elicited by psychedelic compounds, notably the serotonin 5-HT_2A_ agonists lysergic acid diethylamide (LSD), psilocybin, N,N-Dimethyltryptamine (DMT), and their derivatives. These findings suggest a therapeutic potential of psychedelic compounds for some of the behavioural traits associated with autism spectrum disorder (ASD), a neurodevelopmental condition characterized by atypical social behaviour. In this review, we highlight evidence suggesting that psychedelics may potentially ameliorate some of the behavioural atypicalities of ASD, including reduced social behaviour and highly co-occurring anxiety and depression. Next, we discuss dysregulated neurobiological systems in ASD and how they may underlie or potentially limit the therapeutic effects of psychedelics. These phenomena include: 1) synaptic function, 2) serotonergic signaling, 3) prefrontal cortex activity, and 4) thalamocortical signaling. Lastly, we discuss clinical studies from the 1960s and 70s that assessed the use of psychedelics in the treatment of children with ASD. We highlight the positive behavioural outcomes of these studies, including enhanced mood and social behaviour, as well as the adverse effects of these trials, including increases in aggressive behaviour and dissociative and psychotic states. Despite preliminary evidence, further studies are needed to determine whether the benefits of psychedelic treatment in ASD outweigh the risks associated with the use of these compounds in this population, and if the 5-HT_2A_ receptor may represent a target for social-behavioural disorders.

## Introduction

Autism spectrum disorder (ASD) is a neurodevelopmental condition affecting 1–2% of the global population (Chiarotti and Venerosi, 2020). ASD is often diagnosed in childhood, with individuals displaying characteristic atypicalities in social communication and interaction, as well as repetitive patterns of behaviour and restricted interests (American Psychiatric Association, 2013). These features are highly heterogeneous in ASD and are often accompanied with co-occurring diagnoses of depression and anxiety (Rai et al., 2018; Hollocks et al., 2019). At present, there is a lack of selective medications targeting the major phenotypes of ASD: impaired social behaviour and communication.

Psychedelics are currently experiencing a resurgence of scientific investigation, following in the footsteps of pioneering mid-twentieth century research. Although the term “psychedelic” encompasses a variety of compounds, the present review focuses on the serotonergic, or “classical,” psychedelics, which produce their hallucinogenic effects via the serotonin 5-HT_2A_ receptor (De Gregorio et al., 2016a; Holze et al., 2021b; Inserra et al., 2021a). These include lysergic acid diethylamide (LSD), psilocybin, N,N-dimethyltryptamine (DMT), and their derivatives (which will be hereafter referred to as “psychedelics”). Other non-serotonergic psychedelics, such as the empathogen 3,4-Methylenedioxymethamphetamine (MDMA), have also been shown to increase social behaviour and empathy (Bedi et al., 2010; Hysek et al., 2014; Heifets and Malenka, 2016), and to reduce social anxiety in individuals with ASD (Danforth et al., 2018). However, due to MDMA’s vastly different pharmacological properties from serotonergic psychedelics, it will not be discussed in the present review.

Recent clinical and preclinical research demonstrates that psychedelics may hold therapeutic value in the treatment of some of ASD’s core features. Despite the emergence of compelling research, early clinical trials carried out in the 1960s and 70s revealed a variety of side effects after psychedelics were administered experimentally to children with ASD. Thus, the risks associated with the use of these compounds must be carefully examined when considering their potential use in neuroatypical individuals.

## Core Behavioural Atypicalities and Co-Occuring Conditions

ASD diagnoses are contingent on atypicalities in social behaviour. Clinical manifestations include a preference for non-social stimuli (Gale et al., 2019), aberrant non-verbal social behaviours (Osterling et al., 2002), and decreased attention to social stimuli (Sasson and Touchstone, 2014). Despite this diagnostic criterion, no selective treatments for ASD target these core traits. Instead, antipsychotics, antidepressants, mood stabilizers, and stimulants are used to target ASD-associated features, such as irritability, anxiety, and depression (DeFilippis and Wagner, 2016).

It is increasingly apparent that psychedelics enhance social behaviour and elicit empathogenic effects in healthy individuals (see Table 1 for a summarized list of recent clinical trials assessing the use of psychedelics in individuals without ASD). For instance, two psilocybin therapy sessions increased extraversion and openness for up to 3 months in individuals with treatment-resistant depression (Erritzoe et al., 2018). Similarly, a single administration of LSD enhanced sociability and the desire to be with others, while also increasing feelings of trust, closeness, and empathy (Dolder et al., 2016). Another recent study demonstrated that LSD acutely increases emotional empathy and blood levels of oxytocin, a neuropeptide implicated in social behaviour (Churchland and Winkielman, 2012; Holze et al., 2021a). These results have also been corroborated by preclinical evidence, which demonstrate that both acute (Vesuna et al., 2020) and repeated LSD (De Gregorio et al., 2021) enhanced social behaviour in mice.

**TABLE 1:** Recent (2008–2021) clinical trials assessing social behaviour-related effects of psychedelics in neurotypical (non-ASD) individuals

Year	Title	Cohort	Design	Compound	Regimen (dose, frequency, route of administration)	Main outcomes	Side effects	Ref
2008	Mystical-type experiences occasioned by psilocybin mediate the attribution of personal meaning and spiritual significance 14 months later	Healthy volunteers with regular participation in religious/spiritual activities	Randomized, double-blind, placebo-controlled, within-subject	Psilocybin	30 mg/70 kg once, twice or three times	Psilocybin increases altruistic/positive social effects at 14 months follow-up	None reported	Griffiths et al. (2008)
2009	A six-month prospective evaluation of personality traits, psychiatric symptoms and quality of life in ayahuasca-naïve subjects	Volunteers participating in religious Ayahuasca rituals (18–57 y/o, average 35.7 y/o, *n* = 23, 8 males, 15 females)	Observational naturalistic study	Ayahuasca	Up to 12 times over 6 months	Regular ayahuasca users have higher “social functioning” scores in the Short Form-36 Health Survey Questionnaire Ayahuasca might lower social reward dependence by decreasing sensitivity to signals of social approval	None reported	Barbosa et al. (2009)
2011	Psilocybin occasioned mystical-type experiences: immediate and persisting dose-related effects	Healthy volunteers (29–62 y/o average 46 y/o, *n* = 18, 8 males, 10 females)	Randomized, double-blind, placebo-controlled, within-subject	Psilocybin	0, 5, 10, 20, 30 mg/70 kg, per oral solution	Psilocybin increases altruistic/positive social effects at all doses tested	Psilocybin induces dose-dependent acute anxiety/fear 44% of volunteers report delusions or paranoid thinking sometime during the session (especially at 30 mg/70 kg). Effects managed with reassurance in the supportive setting	Griffiths et al. (2011)
						The increase in altruistic/positive social effects elicited by psilocybin 20–30 mg/70 kg are greater than those elicited by psilocybin (0.5–10 mg/70 kg)		
						The increase elicited by psilocybin (20–30 mg/70 kg) in altruistic/positive social effects remains present at 14 months follow-up		
						Volunteers reported better social relationships with family and others		
2015	Acute effects of lysergic acid diethylamide in healthy subjects	Healthy volunteer (25–51 y/o, average 28.6 y/o, *n* = 16, 8 males, 8 females)	Randomized, double-blind, placebo-controlled, within-subject	LSD	200 μg, once, gelatin capsules	LSD increases ratings of “empathogenic drug effects” such as “closeness to others,” “openness,” and “trust” LSD increases circulating oxytocin level	No severe acute side effects	Schmid et al. (2015)
							Most frequent acute or sub-acute mild side effects (up to 72 h): difficulty concentrating, headache, exhaustion, dizziness, lack of appetite, dry mouth, imbalance, nausea	
2016	LSD Acutely Impairs Fear Recognition and Enhances Emotional Empathy and Sociality	Healthy volunteer and mostly hallucinogen-naive (25–65 y/o) volunteers, *n* = 40, 20 males, 20 females)	Randomized, double-blind, placebo-controlled, within-subject	LSD	100 μg, once, per oral solution	LSD enhances explicit and implicit emotional empathy LSD increases prosocial behaviour LSD enhances the desire to be with other people LSD decreases cognitive empathy	None reported	Dolder et al. (2016)
					200 μg, once, per oral solution			
2016	Effects of serotonin 2A/1A receptor stimulation on social exclusion processing	Healthy volunteers (20–37 y/o, average 26.48, *n* = 21, 12 males, 9 females)	Randomized, double-blind, placebo-controlled, within-subject	Psilocybin	0.215 mg/kg, once, per oral solution	Psilocybin reduces the feeling of social rejection Psilocybin decreases the neural responses to social exclusion in the dorsal anterior cingulate cortex and the middle frontal gyrus	None reported	Preller et al. (2016)
2016	Rapid and sustained symptom reduction following psilocybin treatment for anxiety and depression in patients with life-threatening cancer: a randomized controlled trial	Individuals with advanced (stage III or IV) cancer (*n* = 29)	Randomized, double-blind, placebo-controlled, within-subject	Psilocybin	0.3 mg/kg, per oral solution, once	Psilocybin immediately reduced anxiety and depression symptoms for up to 7 weeks post-treatment	None reported	Ross et al. (2016)
						Psilocybin decreased cancer-related demoralization and hopelessness while increasing spiritual well-being and quality of life for up to 6.5 months post-treatment		
						Psilocybin produced positive social effects (ex: increased altruism), positive mood changes and positive changes on attitudes about life and self		
2016	Psilocybin produces substantial and sustained decreases in depression and anxiety in patients with life-threatening cancer: A randomized double-blind trial	Individuals with a life-threatening cancer diagnosis (average age: 56.3 y/o, *n* = 51, 26 males, 25 females)	Randomized, double-blind, crossover	Psilocybin	Low dose: 1 or 3 mg/70 kg High dose: 22 or 30 mg/70 kg Either low or high dose was administered, then after 5 weeks the other dosage was administered	High-dose psilocybin elicited a clinician- and self-rated decrease in anxiety (including death anxiety) and depressed mood, and an increase in quality of life and optimism Effects were still present in >80% of individuals at a 6-months follow-up evaluation	No serious adverse effects	Griffiths et al. (2016)
							Some minor adverse effects (such as psychological discomfort, elevated blood pressure, nausea) occurred during the psilocybin session	
2017	Effect of Psilocybin on Empathy and Moral Decision-Making	Healthy volunteers (20–38 y/o, average 26.72 y/o, *n* = 32, 17 males, 15 female)	Randomized, double-blind, placebo-controlled, within-subject	Psilocybin	Psilocybin 0.215 mg/kg, per oral solution	Psilocybin increases explicit and implicit emotional empathy compared with placebo Psilocybin does not modify cognitive empathy compared to placebo	None reported	Pokorny et al. (2017)
2017	Psilocybin-occasioned mystical-type experience in combination with meditation and other spiritual practices produces enduring positive changes in psychological functioning and in trait measures of prosocial attitudes and behaviours	Healthy volunteers, support for spiritual practice (*n* = 75)	Randomized, double-blind, placebo-controlled	Psilocybin	Very low dose - Psilocybin 1 mg/70 kg (0.0143 mg/kg) per oral solution, twice 1 month apart *n* = 25 High dose - Psilocybin 20 and 30 mg/70 kg (0.29 mg/kg and 0.43 mg/kg) respectively in session 1 and 2, 1 month apart (+standard support for spiritual practice, *n* = 25, + high support for spiritual practice, *n* = 25)	Individuals receiving high dose psilocybin score higher for altruistic/positive social effects than those receiving low dose psilocybin	None reported	Griffiths et al. (2018)
						Individuals receiving high dose psilocybin + high support for spiritual practice score higher for altruistic/positive social effects acutely than dose receiving high dose psilocybin + standard support		
						At 6 months follow-up, individuals who received high dose psilocybin score higher for altruistic/positive social effects than those receiving low dose psilocybin on altruistic/positive social effects		
						At 6 months follow-up, individuals receiving high dose psilocybin + high support for spiritual practice score higher than those receiving high dose + standard support on altruistic/positive social effects		
2017	Long-term follow-up of psilocybin-facilitated smoking cessation	Individuals with tobacco addiction (average 51 y/o, *n* = 15, 10 males, 5 females)	Open label pilot study (follow-up)	Psilocybin	20 and 30 mg/70 kg, two-three times	Altruistic/positive social effects scores remain significantly higher compared to baseline at 12 months follow-up after the first administration	Physiological adverse effects limited to mild post-session headache, and modest acute elevations in blood pressure and heart rate Some participants experienced challenging (fearful, anxiety-provoking) psilocybin session experiences. These effects resolved by the end of the drug session via interpersonal support from study staff	Johnson et al. (2017)
2018	Role of the 5-HT_2A_ Receptor in Self- and Other-Initiated Social Interaction in Lysergic Acid Diethylamide-Induced States: A Pharmacological fMRI Study	Healthy volunteers (20–34 y/o, mean age 25.42 y/o, *n* = 24, 18 males, 6 females)	Randomized, double-blind, placebo-controlled, within-subject	LSD	LSD 100 μg, once, per oral solution	LSD loosens self-boundaries, reducing neural response to self- versus other-initiated real-time social interaction LSD’s reduction in brain activity in regions implicated in self-processing and social cognition was correlated with subjective drug effects LSD alters joint attention processing in the mPFC	No serious adverse events Transient mild headaches after drug effects wore off (*n* = 4 participants) Transient sleep disturbances for the first 2 nights after drug administration (*n* = 1 participant)	Preller, Schilbach, et al. (2018)
				Ketanserin	Ketanserin 40 mg/kg, per oral solution, once prior to LSD 100 μg	LSD increases positive and negative affect scores	No further side effects reported after 3 months	
						Ketanserin blocked the LSD-induced changes in self-processing and social cognition		
2018	Long-lasting subjective effects of LSD in normal subjects	Healthy volunteers (*n* = 16)	Randomized, double-blind, placebo-controlled, within-subject (follow-up)	LSD	200 μg, once, per oral solution	LSD increases ratings of altruistic/positive social effects after 1 and 12 months	None reported	Schmid and Liechti, (2018)
2019	Psilocybin and MDMA reduce costly punishment in the Ultimatum Game	Healthy volunteers (male, *n* = 20)	Open-label, within-participant design	Psilocybin	2 mg per intravenous infusion over 2 min (test performed 60 min later)	Psilocybin increases one’s concern for the outcome of interacting partners	None reported	Gabay et al. (2018)
2019	Acute subjective and behavioural effects of microdoses of LSD in healthy human volunteers	Healthy volunteers (18–40 y/o, *n* = 20, 8 males, 12 females)	Randomized, double-blind, placebo-controlled, within-subject	LSD	LSD 6.5, 13, or 26 μg, per oral solution with tartaric acid (0.5 ml, sublingual)	Dose-related subjective drug effects LSD (26 µg) increased vigor and marginally decreased the positivity ratings for positive pictures	Trend towards increased anxiety with 26 µg dose	Bershad et al. (2019)
						No other effects on mood, cognition or physiological measures		
2019	Replication and extension of a model predicting response to psilocybin	183 valid responses (*n* = 97 males, *n* = 85 females). Average age 31.9 y/o, range 18–70 years	Retrospective survey	Psilocybin (dried mushrooms in pieces, dried powdered mushrooms, fresh mushrooms, synthesized psilocybin) Other unprescribed (such as cannabis, opiates, alcohol, stimulants), and prescribed (antidepressant, anxiolytic, blood pressure medications) substances ingested by some of the respondents	Various amounts ingested in the previous 12 months	Having a complete mystical experience is associated with higher post-treatment scores of empathy and social concern Surrender and preoccupation are the psychological states that produce the greatest (respectively positive and negative) responses	None reported	Russ et al. (2019)
2019	Sub-Acute Effects of Psilocybin on Empathy, Creative Thinking, and Subjective Well-Being	Volunteers attending a psilocybin retreat (average 34.8 y/o, *n* = 55, 24 males, 26 females)	Observational naturalistic study	Psilocybin-containing truffles	34.2 g average of psilocybin-containing truffles in tea form, once. Psilocybin content 0.127 mg/G of truffle (content in 34.2 g = 4.34 mg psilocybin) Psilocin—0.7 mg/G of truffle (content in 34.2 g = 23.9 mg psilocin) Truffles crushed and boiling hot ginger tea added. Truffles remains in the cup eaten optionally	Increased emotional empathy (concern for faces depicting negative emotions) sub-acutely (the morning after) but not after 7 days	None reported	Mason et al. (2019)
						Increased implicit arousal in response to faces depicting positive and negative emotional content the morning after Increased implicit arousal in response to faces depicting negative but not positive emotional content after 7 days		
2019	Exploring ayahuasca-assisted therapy for addiction: A qualitative analysis of preliminary findings among an Indigenous community in Canada	Indigenous members of a rural Coast Salish community in British Columbia (BC), Canada (19–56 y/o, mean 38 y/o, *n* = 11, 6 males and five females)	Observational naturalistic study	Ayahuasca	Ayahuasca (50–100 ml, twice over 2 days)	Some of the participants reported improved emotional openness at 6 months follow up Some of the participants improved their social relationships and had better communication with friends and family at 6 months follow-up	None reported	Argento et al. (2019)
2020	LSD-induced increases in social adaptation to opinions similar to one’s own are associated with stimulation of serotonin receptors	Healthy volunteers (male and female, 20 y/o to 40 y/o, average 25.25 y/o, *n* = 24, 18 males, 6 females)	Double blind, placebo controlled, within subjects	LSD ketanserin	LSD 100 μg, once, per oral solution ketanserin pretreatment (60 min prior) 40 mg, once, per oral solution	LSD increases the adaptation to the opinions of others if they are similar to one’s own LSD modulates neuronal activity in the mPFC during social feedback processing and not during social decision-making (blocked by ketanserine)	No substantial side effects acutely and after 3 months Transient mild headaches (4 participants) after drug effects had worn off Transient sleep disturbances for the first two nights after drug administration (1 participant)	Duerler et al. (2020)
						The magnitude of LSD-induced social adaptation change is associated with personality Patients with higher neuroticism scores adapt more strongly after LSD administration LSD has the strongest impact on social cognition in patients with lower sociability and higher neuroticism scores at baseline		
2021	Role of the 5-HT2A Receptor in Acute Effects of LSD on Empathy and Circulating Oxytocin	Healthy volunteers (25–52 y/o, average age: 29 *n* = 16, 8 males, 8 females)	Double-blind, placeo-controlled, crossover design	LSD ketanserin	LSD 25, 50, 100, or 200 μg, once, per oral solution ketanserin 20 mg, once, per oral solution	LSD dose-dependently increased explicit and implicit emotional empathy LSD 200 µg significantly increased emotional empathy compared to placebo ketanserin did not significantly reduce the LSD-induced increase in empathy LSD increased blood oxytocin levels 1 and 3 h after drug intake, and this effect was blocked by ketanserin administration	None reported	Holze, Avedisian, et al. (2021)

ASD is often accompanied by depression, generalized anxiety, and social anxiety in particular (Spain et al., 2018; Hollocks et al., 2019; Kirsch et al., 2020). These co-occurring diagnoses may also be potential targets of psychedelics. For instance, LSD (Gasser et al., 2014) and psilocybin (Griffiths et al., 2016) have been shown to reduce symptoms of anxiety and depression in patients with life-threatening conditions. Importantly, the anxiolytic effect produced by LSD was appreciable after only two psychedelic-assisted therapy sessions and lasted for up to 12 months without any serious adverse effects; while the attenuative effects of psilocybin occurred after only one dose and were still present 6-months post-administration. Likewise, psilocybin has been shown to reduce depressive symptoms in those with treatment-resistant depression for up to 6 months (Carhart-Harris et al., 2018; Davis et al., 2021). The antidepressant and anxiolytic properties of DMT and ayahuasca have been observed similarly both clinically (Osório Fde et al., 2015; Palhano-Fontes et al., 2019) and pre-clinically (Cameron et al., 2019). It cannot be ruled out that the prosocial effects of psychedelics may reflect their anxiolytic effects, notably with regard to social anxiety.

Further, a recent double-blind randomized trial found that the antidepressant effects of psilocybin were not significantly different than those of the selective serotonin reuptake inhibitor (SSRI) escitalpram (Carhart-Harris et al., 2021). This result, in addition to the lack of a placebo-controlled group, limits support for the efficacy of psilocybin and highlights the importance of assessing whether or not the relative benefits of psychedelics (compared to established medications) warrant their potential side effects.

Despite ongoing research, there is still a lack of systematic, double-blind, placebo-controlled clinical trials assessing the specific therapeutic and adverse effects of psychedelics in neurotypical individuals and in those with ASD. Thus, further research is needed to identify an optimal dose that both minimizes the risk of adverse effects, and importantly, to elucidate whether or not the therapeutic effects of psychedelics observed in neurotypical individuals can be clinically observed in those with ASD. Further research is also needed to better understand how the effects and mechanisms of action associated with psychedelics differ when administered acutely or chronically, and to what extent such interventions provide therapeutic effects for people with ASD.

## Neurobiology

Due to the heterogeneity of ASD, the neurobiological underpinnings of its behavioural phenotypes remain difficult to characterize. ASD is diagnosed according to the criteria of the Diagnostic and Statistical Manual of Mental Disorders, Fifth Edition, including individuals with genetic and non-genetic etiologies across the spectrum. This presents an inherent challenge in hypothesizing how individuals with ASD might respond to psychedelic administration, given that ASD diagnoses cover a large spectrum of neurobiological and genetic profiles.

It is important to note that due to the lack of recent studies assessing the use of psychedelics in ASD, the following discussion reflects neurobiological processes that represent potential targets of psychedelics in ASD, not phenomena which have been proven to be associated with their effects in this population.

### Synaptic Function

Many genes associated with ASD play integral roles in synaptic function (Guang et al., 2018), suggesting a critical involvement of synaptic dysfunction in ASD pathogenesis. Mutations in the SH3 and Multiple Ankyrin Repeat Domains (*SHANK3*) gene—encoding a synaptic scaffolding protein—can cause ASD (Durand et al., 2007). In a transgenic mouse model, this mutation produced a lengthening of dendritic spines and long-term potentiation deficits in the hippocampus (Wang et al., 2011). Mutations in the Contactin-Associated Protein-Like 2 (*CNTNAP2*) gene can also result in ASD (Penagarikano and Geschwind, 2012). *CNTNAP2* encodes a synaptic cell-adhesion protein, and its deletion leads to altered dendritic arborization, spine development, and global synaptic transmission in mice (Anderson et al., 2012; Lazaro et al., 2019). Hyper-methylation of the Fragile X Mental Retardation 1 (*FMR1*) gene—which encodes an important regulatory protein for dendritic mRNA—is an epigenetic modification that can cause ASD (Bassell and Warren, 2008). *FMR1* knockout rats display decreased hippocampal long-term potentiation and long-term depression, in addition to impaired *α*-amino-3-hydroxy-5-methyl-4-isoxazolepropionic acid (AMPA) receptor-mediated synaptic strength (Tian et al., 2017). Recently, ASD-induced synaptic impairments have also been identified in human-derived samples. Induced pluripotent stem cell-derived neurons from individuals with ASD exhibited reduced miniature excitatory post-synaptic current frequency and impaired N-Methyl-D-aspartic acid (NMDA) receptor function (Ross et al., 2020).

Given that alterations in synaptic properties are consistently found in different mouse models of ASD and recently in humans, the ability of psychedelics to modulate synaptic events might play an important role in their potential therapeutic effects in ASD. Recently, a single administration of psilocybin in mice was shown to produce AMPA receptor-mediated synaptic strengthening in hippocampal brain slices (Hesselgrave et al., 2021); an effect which may normalize the synaptic dysfunction of *FMR1* knockout rats (Tian et al., 2017). Additionally, LSD and DMT were shown to promote structural and functional neural plasticity in rodent neuronal cultures and in *Drosophila* larvae (Ly et al., 2018). This study demonstrated that the increased dendritic arbor complexity, dendritic spine growth, and synapse formation caused by psychedelics were mediated by the mammalian target of rapamycin (mTOR)- and 5-HT_2A_-signaling. Accordingly, our study revealed that LSD requires intact mTOR signaling in excitatory neurons and intact 5-HT_2A_ neurotransmission in the mPFC to enhance social behaviour (De Gregorio et al., 2021). Given that mTOR (Hoeffer and Klann, 2010; Li et al., 2010) and the 5-HT_2A_ receptor (Barre et al., 2016; Berthoux et al., 2019) play important roles in mediating neuroplastic events, LSD’s ability to increase social behaviour might be contingent on its ability to modulate neuroplasticity.

Despite the pronounced neurotrophic effects of psychedelics, the dysregulation of mTOR and 5-HT_2A_ signaling in individuals with ASD may be a limiting factor for the therapeutic action of psychedelics on synaptic structure and function. For instance, hyperactive mTOR signaling was found in T cells isolated from children with ASD (Onore et al., 2017) and in post-mortem ASD brain samples (Tang et al., 2014). Positron emission tomography (PET) imaging studies have also revealed that individuals with ASD have reduced 5-HT_2A_-binding affinity in various brain regions compared to neurotypical individuals, suggesting a reduced expression of this receptor (Murphy et al., 2006; Oblak et al., 2013; Brandenburg and Blatt, 2019). In agreement, a significant overrepresentation of the G allele in the -1438 A/G polymorphism in the 5-HT_2A_ gene was found in individuals with ASD (Hranilovic et al., 2010) and this has been associated with decreased receptor expression (Parsons et al., 2004; Myers et al., 2007). Given that mTOR and 5-HT_2A_ mediate psychedelics’ synaptic effects, their dysregulation in ASD might limit or alter the therapeutic effects in these populations.

### Serotonin Signaling

Serotonin (5-HT) is a neurotransmitter and hormone implicated in a variety of physiological phenomena and psychiatric conditions including neuronal development (Brummelte et al., 2017), synaptic plasticity (Kirkwood, 2000), depression (Cowen and Browning, 2015), and ASD (Chugani, 2002). Several lines of evidence suggest a dysregulation of the serotonergic system in ASD. Elevated blood serotonin levels was identified as one of the first putative biomarkers of ASD (Schain and Freedman, 1961), a finding that has been corroborated using meta-analysis, revealing that 28.3% of individuals with ASD have elevated 5-HT levels (Gabriele et al., 2014). Differences in 5-HT production have also been found in the brains of individuals with ASD. For instance, the development of brain serotonin synthesis capacity during childhood is robustly different in children with ASD (Chugani et al., 1999). Human studies generally point to lower levels of brain serotonin in ASD (Adamsen et al., 2014; D'Eufemia et al., 1995; McDougle et al., 1996; Nakamura et al., 2010). However, these studies have used proxy markers of serotonin levels such as PET binding of serotonin transporters and receptors, and cerebrospinal fluid serotonin metabolites. Thus, more direct studies are needed before low brain serotonin can be characterized as a biomarker of ASD. However, in support of this hypothesis are preclinical studies demonstrating that the depletion of brain serotonin in neonatal mice produces ASD-like behaviours such as altered social and stereotypical behaviours and increased anxiety (Boylan et al., 2007; Hohmann et al., 2007).

Interestingly, the 5-HT_1A_ and 5-HT_2A_ receptors may be key mediators of the role that serotonin signaling plays in the pathogenesis of ASD. Neuroimaging studies have found reduced binding affinity of these receptors in limbic and neocortical brain regions of individuals with ASD (Murphy et al., 2006; Oblak et al., 2013; Brandenburg and Blatt, 2019). Accordingly, mice with impaired 5-HT_1A_ or 5-HT_2A_ receptor expression or function display increased anxiety-like behaviour which is rescued with the selective genetic restoration of the respective receptor (Ramboz et al., 1998; Gross et al., 2002; Weisstaub et al., 2006; Piszczek et al., 2015). LSD administration increases brain serotonin levels (Freedman, 1961) and potentiates the excitatory response of 5-HT_2A_ receptor agonism (De Gregorio et al., 2021). Given that psychedelics are agonists of the 5-HT_1A_ and 5-HT_2A_ receptors (De Gregorio et al., 2016a; Inserra et al., 2021a), their pharmacological effects may help restore the altered serotonergic signaling observed in ASD.

Fenfluramine, a serotonin-releasing agent, enhances serotonin signaling in the brain. While few small-sample, placebo-controlled studies found moderate efficacy in fenfluramine’s ability to increase IQ in individuals with ASD (Geller et al., 1982; Ritvo et al., 1984), far more have found that this treatment is only effective in mildy reducing some of the motor and attentional atypicalities in people with ASD. This data suggests that increasing brain serotonin levels (and consequently serotonin signaling) is generally ineffective in improving the behavioural condition of individuals with ASD. Thus, the mechanisms of action of psychedelics must be better characterized in order to assess how they may interact with the altered serotonin signaling observed in ASD.

### Prefrontal Cortex

The prefrontal cortex (PFC), and especially the medial PFC (mPFC), mediates social behaviours and cognition (Grossmann, 2013). Indeed, lesion of the PFC, which is clinically referred to as “frontal lobe syndrome,” induces profound deficits in social interaction (Anderson et al., 1999; Eslinger et al., 2004; Kim et al., 2015; Kirsch et al., 2020). Accordingly, reduced PFC activity is observed in various preclinical models of ASD (Krueger et al., 2011; Duffney et al., 2015; Brumback et al., 2018). Aberrant mPFC activity is also observed in human neuroimaging studies, which report altered mPFC recruitment and connectivity in individuals with ASD compared to neurotypical individuals (Kennedy and Courchesne, 2008; Lombardo et al., 2010; Li et al., 2020). Human post-mortem studies have also found that the PFC of children with ASD have greater neuronal disorganization and differences in neuronal composition compared to neurotypical children (Stoner et al., 2014; Hashemi et al., 2017).

Due to its high 5-HT_2A_ receptor expression, the PFC is highly modulated by the effects of serotonergic psychedelics (De Gregorio et al., 2021; Inserra et al., 2021b; Jakab and Goldman-Rakic, 1998). Indeed, psychedelics activate unique 5-HT_2A_-mediated transcriptional responses in the mouse mPFC (Martin and Nichols, 2016). Corroborating a crucial role of the mPFC in social behaviour, we demonstrated that the photo-inhibition of mPFC excitatory neurons decreases sociability and blocks LSD’s prosocial effects (De Gregorio et al., 2021). Accordingly, in humans, mPFC activation was associated with LSD’s ability to enhance social adaptation to others whose opinions are similar to one’s own (Duerler et al., 2020). Another neuroimaging study revealed that psilocybin dampens mPFC neural activity, an effect correlated with the intensity of the subjective effects (Carhart-Harris et al., 2012). Similarly, LSD reduced the activity of the right mPFC in individuals presented with fearful faces (Mueller et al., 2017).

Although it is not clear which specific biological processes in the PFC may be targeted in ASD by psychedelics, signaling in this brain region plays an important role in the mechanism through which these compounds modulate social behaviour.

### Thalamocortical Circuit

The thalamus plays a significant role in the integration of external and internal stimuli, and its projections to the cerebral cortex are believed to play a vital role in consciousness (Llinas et al., 1998; Redinbaugh et al., 2020). Thus, thalamocortical dysfunction can interfere with complex human behaviours, such as social functioning. Recent studies have employed large, multi-site neuroimaging datasets to assess thalamocortical functional connectivity in ASD.

Given the vast heterogeneity of the ASD spectrum and that some of these studies considered the thalamus with other brain regions as a single subcortical structure in their analysis (Cerliani et al., 2015; Maximo and Kana, 2019), there are some inconsistencies in the literature. Nevertheless, two main connectivity trends are appreciable in individuals with ASD: 1) hyperconnectivity between the thalamus and sensorimotor cortex (Di Martino et al., 2014; Cerliani et al., 2015; Woodward et al., 2017; Maximo and Kana, 2019; Tomasi and Volkow, 2019; Ayub et al., 2021), suggesting an anomalous filtering of sensory information; and 2) hypoconnectivity between the thalamus and multimodal association cortices (Nair et al., 2013; Chen et al., 2016; Maximo and Kana, 2019), suggesting an aberrant integration of sensory information.

The effect of psychedelics on functional thalamocortical connectivity in humans has recently been investigated. Some studies demonstrate a general 5-HT_2A_-mediated increase in thalamocortical connectivity following LSD (Tagliazucchi et al., 2016; Müller et al., 2017), while others show that psychedelics increase or decrease thalamocortical connectivity depending on the cortical region observed (Preller et al., 2018; Preller et al., 2019). Specifically, Preller and others (2018) revealed that LSD increases thalamic connectivity to cortical sensory regions while decreasing its connectivity to associative areas. This specific finding suggests that LSD may potentially exacerbate the abnormal thalamocortical connectivity in individuals with ASD. Thus, more investigation is required to elucidate the link between thalamocortical connectivity and social behaviour in ASD, and the way that psychedelics mediate this link.

In addition to thalamocortical connectivity, the effects of psychedelics on social behaviour may involve the regulation of spontaneous firing of thalamic neurons. The mediodorsal nucleus of the thalamus (MDT) has extensive reciprocal projections to the mPFC and is implicated in various cognitive functions, including sociability (Ferguson and Gao, 2018; Parnaudeau et al., 2018). Individuals with ASD present with morphological thalamic alterations, such as decreased global thalamic volume (Tsatsanis et al., 2003; Waiter et al., 2004), and increased surface area of the MDT specifically (Schuetze et al., 2016). Interestingly, the pharmacogenetic inhibition of MDT projections has been shown to reduce social preference in rats (Ferguson and Gao, 2018), further supporting this nucleus’s role in mediating social behaviour. We recently discovered that LSD increases neuronal firing in the MDT (Inserra et al., 2021b), suggesting that LSD’s effects may partly be mediated by its modulation of MDT projections.

Altogether, we suggest that psychedelics may target the dysregulated thalamocortical connectivity and thalamic neuronal firing in individuals with ASD. However, it is also true that the thalamocortical dysregulations in individuals with ASD may concurrently limit the behavioural effects of psychedelics (such as changes in social behaviour) that are mediated by thalamocortical signaling. For instance, mice with embryonic-stage deletions of the Tuberous Sclerosis Complex 1 (*Tsc1*) gene (a standard preclinical model of ASD) had an overabundance and greater diffusion of thalamic projections to the somatosensory cortex (Normand et al., 2013). Since structural thalamocortical connectivity is profoundly altered in this ASD model, the response of this circuit to the administration of psychedelics may also be altered. Thus, any cognitive effects of psychedelics mediated by their ability to modulate thalamocortical signaling may be significantly different in people with ASD, potentially limiting their therapeutic effects.

## Clinical Trials of Psychedelics in Children With ASD (1961–1970)

Prior to the classification of psychedelics as Schedule 1 Controlled Substances in 1970, these substances were tested in the treatment of children with ASD in order to assess their efficacy in relieving treatment-refractory ASD-like behaviours. Importantly, these individuals were classified as “autistic-schizophrenic” (Bender et al., 1961; Freedman et al., 1962; Bender et al., 1966; Fisher, 1970), and “severely emotionally disturbed” (Fisher and Castile, 1963). Thus, they may not have necessarily been diagnosed with ASD using contemporary diagnostic criteria, imposing a significant limitation on these findings (reviewed and summarized in Table 2). Although significant methodological and ethical shortcomings are evident through the lens of modern clinical and ethical research standards, this early work is being re-scrutinized to extrapolate potentially meaningful data which could inform contemporary research.

**TABLE 2:** Early clinical trials (1961–1970) assessing the use of psychedelics in “autistic schizophrenic” children. The terms “autistic” and “schizophrenic” do not reflect the currently approved terminology of the DSM-V, but rather the terminology that was used at the time of these early studies.

Year	Title	Cohort	Design	Compound	Regimen (dose, frequency, route of administration)	Main outcomes	Side effects	Ref
1961	Treatment of Autistic Schizophrenic Children with LSD-25 and UML-491	Hospitalized “autistic-schizophrenic” children (*n* = 14, 11 boys, 3 girls, 6–10.5 years old)	Open label	LSD	LSD	LSD	LSD	Bender et al. (1961)
				L-methyl-D-Iysergic acid butanolamide (UML-491)	Acute–25 μg, intramuscular	Acute–Improved behavioural patterns. Children unusually interested in the surrounding environment and seeking physical interaction. Children engaged in playful activities such as hand clapping and body swaying. Decreased appetite	No severe side effects reported. 2 prepuberal (10-year-old) children displayed disturbed anxious behaviour and were dropped from the study UML-491	
					Repeated–100 μg, per oral solution, once per week in the early morning			
					Subsequently increased to 100 µg per oral solution 2 to 3 times per week	Repeated - When given 2–3 times per week, effects persist: decreased aggressive behaviour towards peers, increased interaction-seeking and emotional and physical closeness with peers and adults, more spontaneously playful, improvements in physical condition, “rosy” color rather than “blue” or “gray” and increased weight gain, enhanced understanding of environmental stimuli and more appropriate reactions to them, higher maturity in the Vineland Maturity Scale rating. Increased inorganic phosphate blood level. Changes appear to become chronic with continuous administration of the drug UML-491	Motor restlessness, irritability, localized muscle tensions or spasms, mild “crawling” skin sensations	
					Finally, 100 µg per oral solution daily for 6 weeks UML-491,8 mg divided in 4 doses daily			
					Other pharmacological treatments interrupted at the onset of treatment with LSD and UML-491	Enhanced mood, relief of episodic headaches, relief of perceptual hypersensitivity in visual, auditory, olfactory, and skin sensations, general sense of wellbeing with improved sleep patterns	Episodes of changing muscle tensions and kinesthetic sensations with clowning, staggering gait, and twisting of the neck, back, and arms	
1962	Autistic Schizophrenic Children. An Experiment in the Use of D-Lysergic Acid Diethylamide (LSD-25)	“Autistic-schizophrenic” children (*n* = 12, 10 boys, 2 girls, aged 5 years 11 months to 11 years 10 months)	Open label	LSD	100 µg (1 girl 50 μg, 1 boy 200 µg), per oral solution, once or twice. Other Pharmacological treatments (unspecified tranquillizers, interrupted 24 h prior to LSD treatment)	Increased body awareness	Sharp and rapid mood swings Severe anxiety (4 children) Moderate anxiety (3 children) Panic-like state (1 child) Children seemed to be experiencing auditory and visual (the latter more predominant) hallucinations Catatonia (3 children)	Freedman et al. (1962)
						Desire for increased physical contact Repetitive behaviours disappeared and then reappeared as LSD wore off Two of the non-verbal children seemed to experiment with new sounds	Acute ataxia (1 children)	
						Decreased appetite	Acute mild ataxia (4 children)	
1963	Interim report on Research project: An Investigation to Determine Therapeutic Effectiveness of LSD-25 and Psilocybin on Hospitalized Severely Emotionally Disturbed Children	Hospitalized Severely Emotionally Disturbed Children (*n* = 12, 4 y 10 m—12 y 11 m, average 9 y 10 m)	Unknown	LSD Psilocybin	LSD alone (50–400 μg, typical dosage 200–300 µg)	Increased sociability Increased face gazing Decreased anger outbursts	2 episodes of seizures (1 girl) developed during treatment which was discontinued in this child	Fisher and Castile, (1963)
					Psilocybin alone (10–20 mg, typical dosage 14–16 mg) LSD (100–300 μg, typical dosage 200 µg) + psilocybin (10 mg)	Increased parent relatedness		
					Librium (10 mg) and Methedrine (5 mg) were used as pre-treatment medication in some sessions	Increased relatedness to peers and adults		
					“3 grains of dilantin” administered the night before treatment to reduce the likelihood of seizures	Increased desire to communicate		
					“Boosting” dose (LSD 25–100 µg) available if the child seemed to be a) caught in a problem or area that he could not break through, b) regressing to psychotic and stereotyped behaviour, c) defending themselves from new experiences	Decreased anxiety and compulsive behaviour for about 1 day following the treatments		
					1–11 treatments, every 14 days–1 month	1 self-harming girl who was in continuous restraint to prevent fatal self-harm stopped self-harming, did not need restraints anymore and became toilet-trained Children who respond better		
						- Have speech		
						- Are more “schizophrenic” than “autistic”		
						- Are older (10–12 y/o)		
1963	LSD and UML treatment of hospitalized disturbed children	Hospitalized children (*n* = 50). Half displayed autism spectrum disorder features and half displayed psychotic features	Open label	LSD	LSD gradually increased from 50 to 150 µg daily, divided in two doses UML gradually increased from 4 to 12 mg daily, divided in two doses	Increased responsiveness to environmental stimuli	No serious adverse events	Bender et al. (1963)
				UML	Daily administration, duration of treatment: 2–12 months	Increased alertness, awareness, and affectionate behaviour	Some of the quieter “autistic” children became mildly aggressive	
						Some of the more aggressive children became remarkably quieter after LSD treatment		
						Decrease of regressive behaviour in some “autistic” children		
						Improved food habits and interest in new foods		
						Improved vocabulary in some of the children		
						After taking the children off LSD their behaviour regressed but to a lesser extent		
1966	Modification of autistic behaviour with LSD-25	“Autistic” twin males (4 years 9 months old at study start, 5 years 2 months at study end)	Double-blind placebo-controlled	LSD	LSD 50 µg per oral solution 9 treatments, twice weekly 50 mg oral. chlorpromazine to terminate the LSD effects after 3 h	Increased eye-to-face contact during LSD sessions	None reported	Simmons et al. (1966)
						Increased responsiveness to adults		
						Increased movements towards the experimenter; decreased movements away from the experimenter		
						Decreased repetitive behaviours during LSD sessions		
						Increased smiling and laughing behaviour		
1966	The Treatment of Childhood Schizophrenia with LSD and UML	“Autistic”, regressed, verbal, psychotic and “schizophrenic” (different groups) pre-and post-puberty male and female children 6–15 years of age (total *n* = 54)	Open label	LSD	LSD	*Prepuberty “autistic” boys*	In “autistic” prepuberal boys, no regressions were observed although some children had episodic occurrences of aggressive contact with other children and feces smearing	Bender et al. (1966)
				UML	100–150 μg, daily in 2 doses UML	All participants show some mild (variable) degree of favorable response with slow and steady progression	One of the “autistic” prepuberal girls became too active and aggressive towards other children (reserpine was administered with LSD to counteract these effects)	
					12 mg daily, divided in 2 doses			
					Duration of treatment: 2–18 months, with an average of 9 months	Participants were happier following the ingestion of the drug and this tended to carry over through the whole day	Some of the “autistic” postpuberal boys attempted to interact with others via biting and pinching	
						Participants became spontaneously more playful with toys, other children and adults		
						Participants sought and responded to physical contact and affection		
						Decreased overall aggressive behaviour		
						Food habits and toilet-training improved in some participants		
						Improved skin color and overall physical health		
						Decreased rhythmic and stereotyped behaviour		
						Increased responsiveness to environmental cues, stimuli and patterns		
						Increased laughing behaviour		
						Increased maturity in the Vineland Social Maturity Scale (the authors suggest that the real improvements are underestimated by this scale)		
						*Prepuberty “autistic” girls*		
						No detectable improvement in maturation or social maturity		
						Two of the three girls seemed brighter and improved in color, weight gain, and eating and toilet habits		
						*Postpuberty “autistic” boys*		
						Similar responses to younger “autistic” children although responses are attenuated compared to prepuberty “autistic” boys		
						Increased typical behaviours such as climbing, ball bouncing and rocking		
						Strong improvement in verbalization and speech appropriateness in one boy		
						For the first time, some children attempted to contact/approach adults		
1970	The Psycholytic Treatment of a Childhood Schizophrenic Girl	Case report (1 of the “autistic-schizophrenic” girls of the clinical trial by Freedman et al., 1962	Open label	LSD Psilocybin	16 treatments over 11 months with LSD alone (50–300 μg, *n* = 13 treatments), psilocybin alone (10–20 mg, *n* = 2 treatments), or LSD (200 µg) + psilocybin (10 mg, *n* = 1 treatment)	Increased motor and verbal behaviour, increased desire for physical contact, the child was delighted and excited with perceptual changes. Decreased repetitive movements. Enhanced sense of humor; she was able to feel and relate to others in a “normal” way	Mood swings including	Fisher, (1970)
					Methedrine (5 mg) and Librium (10–25 mg) given in some instances in combination with LSD (200–300 µg) and psilocybin (10 mg) + LSD (200 µg)	At the end of the treatment she reached a state of deep acceptance and profound feelings of love and personal integration. She ceased her isolated, “autistic” behaviour, talked rationally, and helped other children. Despite scarce follow-up records, she seemed to have stabilized at 5 years follow-up	Anxious behaviour, restless behaviour, angry behaviour, stuffing objects in her mouth	
							Emergence of internal conflict and anger led to anxious, agitated, and self-harming behaviour	
							At the end of treatment 4, she became very violent and tried to choke herself as well as others	

Most of these studies involved a regimen of LSD given at medium to high doses (25–400 µg), with schedules ranging from a single administration to daily administrations for up to 18 months. The most effective results were observed when daily or weekly LSD was given over relatively extended periods of time (Bender et al., 1961). Greater improvements were observed when the therapist was more actively involved with the children; when they were given the possibility to experience meaningful interpersonal psychotherapeutic interactions; and when the settings were free of artificial or experimental restrictions. When the children were taken off the drug, their behaviour regressed, but not to the extent observed previously (Bender et al., 1963).

Psychedelic-assisted therapy in children with ASD resulted in a variety of clinical improvements: enhanced mood, sociability, and affectionate behaviour; increased emotional closeness, relatedness, and responsiveness to others; increased desire to communicate and interest in the surrounding environment; relief of perceptual hypersensitivity; improved speech and vocabulary; increased playfulness, smiling, and laughing; increased eye and face-gazing behaviour; decreased aggressive and repetitive behaviours; and improved sleep patterns.

Although the aforementioned effects of psychedelics are desirable in the treatment of ASD, adverse effects of varying severity were also reported. Some of the children experienced rapid mood swings, ataxia, and moderate to severe anxiety, with at least one case of a “panic-like state” (Bender et al., 1961; Freedman et al., 1962). One girl experienced two episodes of seizures during LSD treatment (Fisher and Castile, 1963). Some of the children displayed increased biting and pinching behaviour, some engaged in aggressive behaviour even after the effects of the drug had worn off, and some had difficulty sleeping in the days following administration (Bender et al., 1961; Freedman et al., 1962; Bender et al., 1963; Fisher and Castile, 1963; Bender et al., 1966; Fisher, 1970). In one “autistic-schizophrenic” girl receiving LSD and psilocybin, the emergence of internal conflict led to acute anxious, aggressive, and self-harming behaviour (Fisher, 1970).

Given that certain individuals with ASD present atypical behavioural characteristics such as increased aggression (Fitzpatrick et al., 2016) and epilepsy (Tuchman and Rapin, 2002), it is not entirely surprising that psychedelic treatment triggered aggressive behaviour (Bender et al., 1966) and seizures (Fisher and Castile, 1963) in some of the children. Consequently, serious precautions must be taken when using psychedelic treatments in these vulnerable populations.

Another potential risk is the potential for psychedelics to induce psychosis and/or schizophrenia. The prevalence of schizophrenia is significantly higher in people with ASD compared to neurotypical individuals (Zheng et al., 2018). Since psychedelic use is associated with the development of psychosis in people with genetic predispositions (Breakey et al., 1974; Vardy and Kay, 1983), the risk of psychosis and schizophrenia must be carefully considered when assessing the potential adverse effects of psychedelic administration in this population. Altogether, although some therapeutic effects of psychedelics in children with ASD have been reported, the extended list of reported adverse effects demands caution.

## Conclusion

Due to the limited treatment options for ASD, the development of novel therapies is warranted. Clinical and preclinical trials suggest that psychedelics may improve social behaviour and decrease the burden of co-occurring diagnoses in ASD by targeting synaptic function, serotonin signaling, PFC activity, and thalamocortical signaling. Early clinical trials in childhood ASD suggest that psychedelics might hold therapeutic potential; however, the side effects encountered represent potential limitations to this treatment. It is possible that psychedelics may alleviate a few core social-behavioural features in individuals with ASD, such as social anxiety, but carefully performing a risk-to-benefit assessment is crucial due to the severity of their potential side effects.

Individuals with ASD represent a highly heterogeneous demographic; therefore, only certain subsets of individuals with ASD may respond well to psychedelic treatment options. Clinical trials must proceed with caution because this population is also comprised of children and some individuals with intellectual disabilities, for which obtaining informed consent is a challenge. Future studies must make these considerations when determining if some of the positive findings obtained in the “first wave” of psychedelic research in ASD can be validated when employing contemporary scientific and ethical standards.
